# Association of Delayed Surgery for Ankle Fractures and Patient-Reported Outcomes

**DOI:** 10.1177/10711007211070540

**Published:** 2022-02-20

**Authors:** Kristian Pilskog, Teresa Brnic Gote, Heid Elin Johannessen Odland, Knut Andreas Fjeldsgaard, Håvard Dale, Eivind Inderhaug, Jonas Meling Fevang

**Affiliations:** 1 Orthopedic department, Haukeland University Hospital, Norway; 2 Clinical Institute 1, The University of Bergen; 3 Department of Physiotherapy, Haukeland University Hospital, Norway

**Keywords:** fracture, ankle, posterior malleolus, complications, PROM, outcome, SEFAS, operation, delay

## Abstract

**Background:**

Several studies probe the association between prolonged time to surgery and postoperative complications in ankle fractures, but little is known about how a longer wait time affects clinical outcomes. The present study aims to assess the association between time from injury to surgery and patient-reported outcomes after operative treatment of severe ankle fractures.

**Method:**

Patients treated operatively for low-energy ankle fractures that also involve the posterior malleolus from 2014 to 2016 were included. Patient charts were reviewed for patient demographics, type of trauma, fracture characteristics, treatment given, and complications. Ankle function was evaluated on a follow-up visit by clinical examination, radiographs, and patient-reported outcome measures (Self-Reported Foot and Ankle Score [SEFAS], RAND-36, visual analog scale [VAS] of Pain, VAS of Satisfaction). We compared patients treated within 1 week to those treated later than a week from injury for analyses.

**Results:**

Follow-up visits of 130 patients were performed at mean 26 (SD 9) months after surgery. Patient demographics and fracture characteristics were similar between groups. Mean SEFAS was 34 (SD 10) in patients treated later than a week from injury vs 38 (SD 9) in those treated earlier (*P* = .012). Patients operated on later than 7 days from injury reported more pain (*P* = .008) and lower satisfaction than those treated earlier (*P* = .016).

**Conclusion:**

In this retrospective patient series of low-energy ankle fractures with posterior malleolar fragments, we found that waiting >7 days for definitive surgery was associated with poorer clinical outcomes and more pain compared with those who had surgery earlier.

**Level of Evidence:**

Level III, retrospective comparative study.

## Introduction

Operative treatment of ankle fractures comes with the risk of various short- and long-term complications, such as soft tissue problems and fracture-related infections (FRIs), malreduction, hardware-related symptoms, pain, and reduced range of motion.^4,5,15,21,23,31^ Timing of surgery and its impact on such complications is an ongoing debate. Schepers et al^26^ found a complication rate of 12.9% in delayed (>6 days from day of injury) ankle fracture surgery. A delay of surgery might be due to delayed admission to hospital, need for additional computed tomography (CT) scans, or more commonly, preoperative soft tissue challenges or scheduled treatment at a later point in time.^2,19,24^ In case of soft tissue challenges, a delay is considered beneficial for the patients as reduced soft tissue swelling might lower the risk of complications.^2^ A temporary external fixator may be applied prior to definitive surgery as immediate care of the injured ankle.^29^ On the other hand, an early and immediate operation might prevent the aforementioned complications and allow early-onset rehabilitation.^25^ Although the association between time from injury to surgery and postoperative complications is well documented, there is a paucity in the literature on any effect from a delay in surgery on postoperative clinical outcomes.^1,25^ The current study therefore aimed to investigate whether a delay from time of injury to definitive operation has an impact on patient-reported outcome after operative treatment of severe ankle fractures compared with earlier surgery.

## Patients and Methods

**Figure 1.**
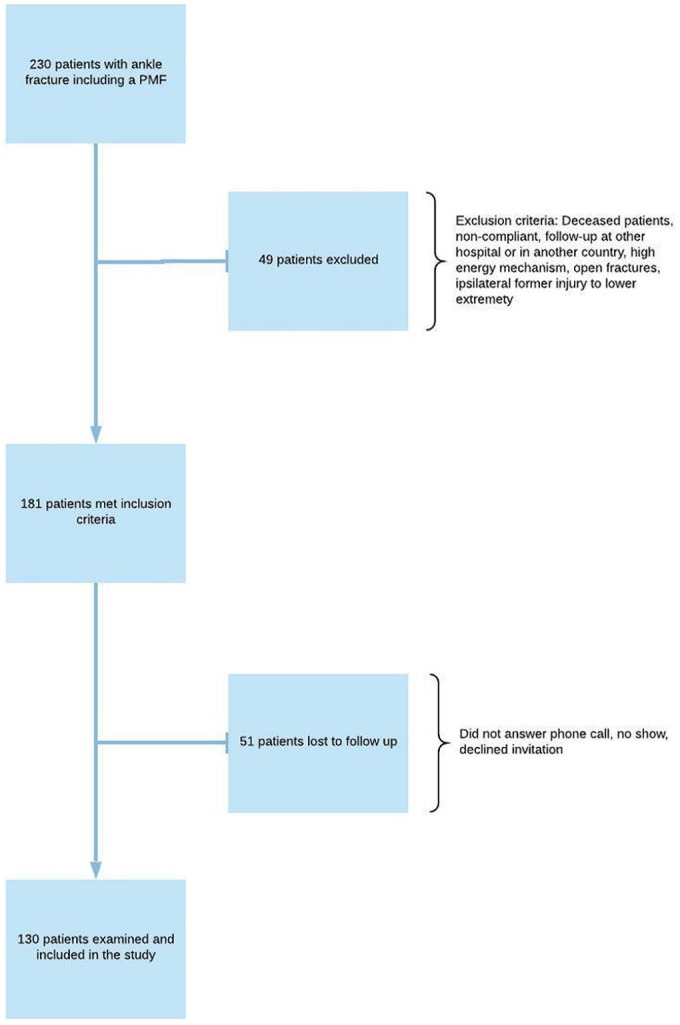
Patient selection, exclusion, and inclusion criteria. PMF, posterior malleolus fracture.

Patients with ankle fractures involving the posterior malleolus treated at Haukeland University hospital in Bergen, Norway, from January 2014 through December 2016 were eligible for the study. Ankle fractures with a posterior malleolus fragment (PMF) are known to have a poor outcome and are therefore defined as “severe ankle fractures” in the current paper.^10,23^ Inclusion and exclusion criteria are presented in Figure 1. Patients were identified by a selective search in the operation planning system (Orbit version 5.11.2, Evry Healthcare Systems AB) on Nordic Medico-Statistical Committee Classification of Surgical Procedures codes for bi- and trimalleolar fractures. Preoperative radiographs were examined, and only patients with ankle fractures that also involved the posterior malleolus were included.^20^ All injuries were low-energy mechanism fractures. Patient charts were reviewed for information concerning patient demographics, type of trauma, fracture characteristics, treatment given, and complications. Eligible patients were invited to a follow-up visit that included clinical examination, radiographs, and patient-reported outcome measures (PROMs)—and the ankle-specific questionnaires thereunder.

The Helse Bergen data protection officer and regional committee for medical and health research ethics (REC) approved the project (REC ID 2016/1720). Informed, signed consent was obtained from all patients prior to inclusion.

The current study assessed if there was a difference in patient-reported outcome between patients treated with definitive surgery within a week from injury (0-7 days) compared with those treated later than a week from injury. To further examine the impact of time for injury to definitive surgery, the patients were stratified based on time from injury to definitive surgery: group 1, within the same day; group 2, within 1-7 days; and group 3, later than 7 days after injury.

### Outcome Assessment

Primary outcome was the Self-Reported Foot and Ankle Score (SEFAS).^6–9,11,12^ SEFAS was translated into Norwegian, and the translation was approved by the Center on Patient Reported Data.^16^ The worst possible score was 0, and the best possible score 48.

Quality of life was assessed using the RAND-36, translated into Norwegian by the Norwegian Institute of Public Health.^14^

Patients scored a visual analog scale (VAS) of Pain from 0 (no pain) to 10 (worst imaginable pain) describing an average of the pain experienced the last 2 weeks before the follow-up appointment. VAS of Satisfaction was rated from 0 (very unsatisfied) to 10 (very satisfied) based on how satisfied the patients were with the result after the injury and the following surgical treatment.

Clinical examination included range of motion (ROM) in passive dorsiflexion and active plantarflexion and heel raise distance for both the operated and the uninjured ankle. Any differences between sides were noted. Positive numbers denote larger movement of the uninjured ankle and negative numbers larger movement of the injured ankle.

Based on chart reviews, complications such as reoperations and revisions, nerve injuries, FRIs,^21^ mechanical irritation from implants, and implant removal were registered. Reoperation was defined as any new surgery associated to the primary open reduction and internal fixation (ORIF), due to malreduction or failed syndesmotic fixation after primary surgery. Revision was defined as surgery performed owing to FRI.

Preoperative radiographs were used to grade fractures according to the Weber classification.^30^ Grade of osteoarthritis (OA) was assessed according to the Kellgren and Lawrence classification from radiographs acquired at follow-up.^18^ Radiographic examination was performed by 2 of the authors, both experienced ankle fracture surgeons (HEJO and KP).

### Surgical Technique

Fractures were treated after standard AO principles. Depending on the size of the PMF, patients were either treated with a traditional approach or a posterior approach. With the traditional approach, the fractures of the lateral and/or medial malleoli were openly reduced and fixed via a direct lateral and medial skin incision. If the size of the PMF was considered to involve 25% or more of the distal tibial articulate surface on lateral radiographs, they were fixed with closed reduction and an anteroposterior screw. Smaller fragments were left unfixed. Patients treated with a posterior approach had the PMF fixed after open reduction with a posterolateral and/or medial approach to the fragment. Fibular fractures were fixed through the same posterolateral incision.

#### Statistics

IBM SPSS version 24 (IBM Corp) and R (CRAN) was used for analyses. SEFAS was assessed both between the group of patients treated within a week vs those treated after a week from injury, and between the 3 stratification groups (definitive surgery at <1 day, 1-7 days, and >7 days from injury). The significance threshold for SEFAS was set at .05. The association of time from injury to definitive surgery on SEFAS was assessed using a linear model while adjusting for age, gender (female), and American Association of Anesthesiologists (ASA) classification. Secondary patient-reported outcomes were tested with a Bonferroni correction at 0.05/3 = 0.017. Continuous variables for the 3 stratification groups were analyzed with the analysis of variance with 2 degrees of freedom and with post hoc Bonferroni and Tukey honestly significant difference tests. One patient did not report their RAND-36 score and another did not report the VAS of Satisfaction score. Categorical variables were analyzed with Pearson chi-squared test, and between-group differences were controlled for with the Bonferroni method for adjusting *P* values while comparing column proportions. Dichotomous variables were analyzed with the Student *t* test for independent variables. The analyses of the reasons for use of external fixation and complications are exploratory and secondary analyses with a threshold of *P* = .05.

## Results

The search rendered 181 patients eligible for inclusion. Of these, a total of 130 patients (72%) were available and met to a follow-up consultation at mean 26 (SD 9) months after surgery. Definitive surgery within a week from injury was performed on 86 patients (66%), and 44 patients (34%) were treated >7 days from injury. Distribution of gender, American Society of Anesthesiologists classification, current smoking status, diabetes mellitus, type of fracture (Weber B or C), rate of dislocation fractures, and use of syndesmotic fixation did not differ between these 2 groups of patients (all with *P* value > .07). However, patients who had definitive surgery after a week from injury more frequently got a temporary external fixator prior to definitive surgery (7/86 patients [8%] vs 34/44 [77%], *P* value *<.*001).

After stratification into 3 groups, there were 44 patients in group 1 (definitive surgery within the same day as the injury), 42 patients in group 2 (definitive surgery within 1-7 days from injury), and 44 patients in group 3 (definitive surgery later than 7 days from injury). Patient demographics and fracture characteristics did not differ between the 3 groups (Table 1).

**Table 1.** Patient Demographics, Fracture Characteristics, Treatment Summary, and Complications Based on Time (Days) From Injury to Definitive Surgery.

	<1 d (n = 44)	1-7 d (n = 42)	>7 d (n = 44)	*P* Value
Patient factors				
Female, n (%)	34 (77)	30 (71)	30 (68)	.6
Age, y, mean (SD)	53 (16)	54 (18)	55 (16)	.9
ASA ≥ 3, n (%)	0 (0)	2 (5)	4 (9)	.1
Diabetes, n (%)	1 (2)	1 (2)	4 (9)	.2
Smoking, n (%)	8 (18)	6 (14)	10 (23)	.6
Fracture characteristics, n (%)				
Weber class B/C	29 (66)/15 (34)	25 (60)/17 (40)	25 (57)/19 (43)	.7
Ankle fracture dislocation	17 (39)	18 (43)	24 (55)	.3
Treatment summary^a^				
Time from injury to operation, d, mean (SD)	0 (0)^b^	4 (2)^c^	12 (3)^d^	<.001
Length of stay, d, mean (SD)	3 (2)^b^	8 (4)^c^	16 (5)^d^	<.001
Postoperative length of stay, d, mean (SD)	3 (2)	3 (3)	4 (4)	.2
Duration of operation, min, mean (SD)	86 (37)	89 (36)	124 (51)^d^	<.001
External fixator, temporary, n (%)	0 (0)^b^	7 (17)^c^	34 (77)^d^	<.001
Syndesmotic fixation, n (%)	31 (71)	21 (50)	20 (46)	.04^e^

Abbreviation: ASA, American Society of Anesthesiologists classification.

a Post hoc analyses for differences between groups were performed with both Tukey honestly significant difference and Bonferroni.

b Statistically significant difference at an alpha level of 0.017 between group 1 and both groups 2 and 3.

c Group 2 differs from groups 1 and 3.

d Group 3 (≥7 days) differs from both groups 1 and 2.

e Post hoc analysis of between-group differences of categorical variables were performed with Bonferroni method for adjusting *P* values while comparing column proportions. Significant difference was found in the use of syndesmotic fixation between group 1 and 3 (*P* = .018), but not between group 1 and 2 or group 2 and 3.

The mean duration of operation was longer in group 3 compared with the 2 other groups. Patients who were treated with temporary external fixator prior to definitive surgery were only found in group 2 (7 of 42 patients, 17%) and group 3 (34 of 44 patients, 77%) (Table 1). The treatment summary in Table 1 shows that mean time from injury to operation and mean length of stay was longer for these patients. The mean time from injury to application of external fixator was 1 day (SD 1) in group 2 and 2 days (SD 2) in group 3 (*P* = .33). The reasons for applying temporal external fixation were similar across groups (Table 2). The exceptions were a higher frequency of unreducible (by cast application) fractures and that the surgeon on call considered it better for the patient to temporarily have the ankle reduced in an external fixator in group 3. Severe soft tissue swelling (33/41 patients) and skin necrosis or blisters in need of healing (8/41 patients) were the main reasons for a delay from application of external fixation until definitive surgery. Among the 59 patients with a dislocation fracture of the ankle, 31 (53%) patients did not get an external fixator, whereas 28 (47%) patients did. Patient and fracture characteristics of patients who did and did not get an external fixator and patients with and without a dislocation fracture are presented in Table 3.

**Table 2.** Reasons for Applying External Fixator.^a^

	<1 d, n (%) (n = 44)	1-7 d, n (%) (n = 42)	>7 d, n (%) (n = 44)	*P* Value^b^
Difficult fracture reduction in the ED	0	4 (10)	14 (32)^c^	.01
Soft tissue swelling and blisters	0	1 (2)	4 (9)	.2
Dislocation of fracture while in cast	0	2 (5)	8 (18)	.04
Considered initially better for soft tissue	0	0	7 (16%)^c^	.01
Skin excoriation at time of injury	0	0	1 (2)	.3

Abbreviation: ED, emergency department.

a A total of 41 patients had an external fixator applied prior to definitive surgery.

b *P* values in the table are calculated with chi-square analyses from a cross-table with 1 degree of freedom comparing group 2 (1-7 days) and group 3 (>7 days).

c Using Bonferroni post hoc analyses, group 3 (>7 days) significantly differs from group 1 and 2. Post hoc analysis does not reveal any significant difference between group 1 and group 2.

**Table 3.** Patient Demographics, Fracture Characteristics, and Treatment Summary for Patients Treated With or Without a Temporary External Fixator, and Patients With and Without a Dislocation Fracture.

				Patients With Dislocation Fracture (n = 59)		
	No Ex-Fix (n = 89)	Ex-Fix (n = 41)	*P* Value	<7 d (n = 35)	>7 d (n = 24)	*P* Value
Patient factors						
Female, n (%)	66 (74%)	28 (68%)	.5	25 (71%)	18 (75%)	.8
Age, y, mean (SD)	53 (17)	57 (16)	.2	55 (17)	56 (13)	.7
ASA ≥ 3, n (%)	2 (2%)	4 (10%)	.06	2 (6%)	2 (8%)	.7
Diabetes, n (%)	4 (5%)	2 (5%)	.9	2 (6%)	3 (13%)	.4
Smoking, n (%)	16 (18%)	8 (20%)	.4	4 (11%)	6 (25%)	.2
Fracture characteristics, n (%)						
Weber class B/C	54 (61%)/	25 (61%)/	>.99	22 (63%)/	14 (58%)/	.7
	35 (49%)	16 (39%)		13 (37%)	10 (42%)	
Dislocation fracture	31 (35%)	28 (68%)	<.001			
External fixator				6 (17%)	22 (92%)	<.001
Treatment summary, mean (SD)						
Time from injury to operation, d	3 (4)	11 (4)	<.001	2 (3)	11 (3)	<.001
Length of stay, d	6 (4)	16 (6)	<.001	6 (4)	16 (5)	<.001
Duration of operation, min	91 (41)	119 (48)	.01	94 (39)	126 (49)	.009

Abbreviations: ASA, American Society of Anesthesiologists classification; Ex-Fix, external fixator.

A posterior approach for surgery was used in 54 (42%) patients whereas the traditional approach was used in 76 (58%) patients. In patients treated later than 7 days from injury, 33 of 44 were treated with a posterior approach (*P< .*001).

### Outcome Evaluation at Follow-up

**Figure 2.**
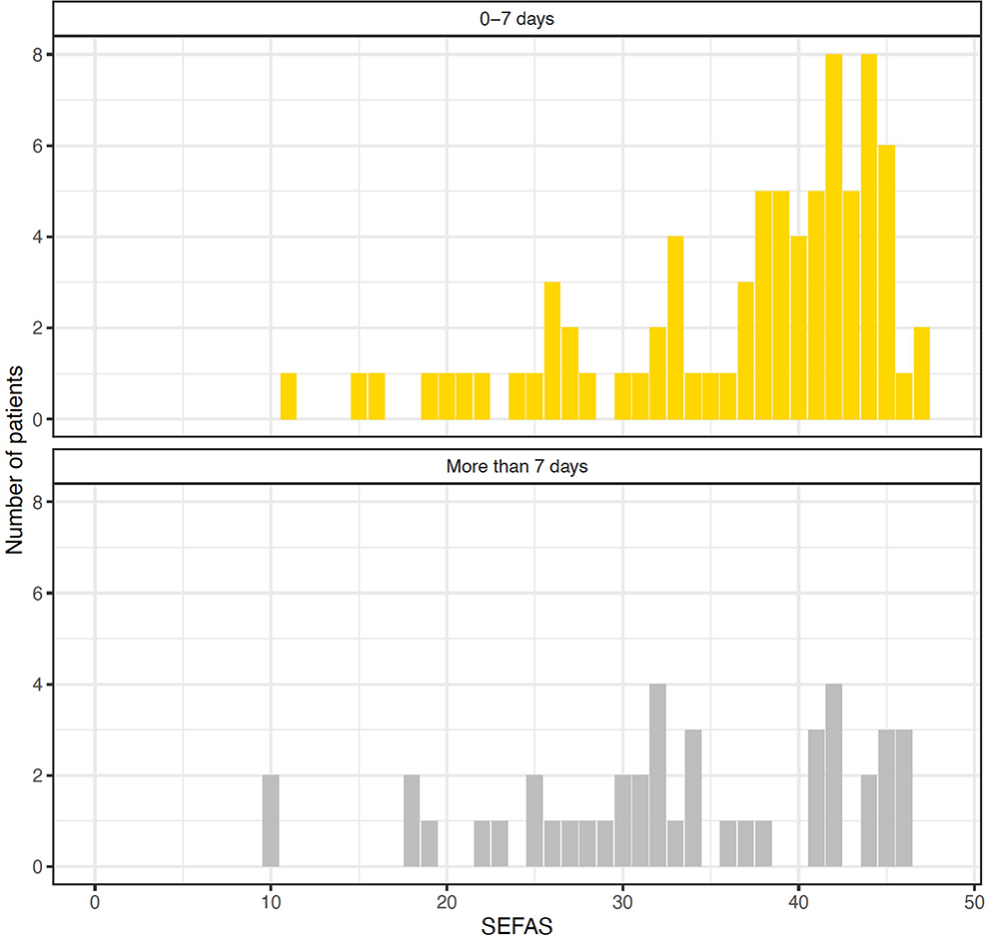
Histograms of the distribution of Self-Reported Foot and Ankle Score (SEFAS) in patients treated within (upper panel) and after (lower panel) a week from injury.

When applying a dichotomous analysis strategy, patients treated later than a week from injury had a lower mean SEFAS, higher VAS of Pain, and lower VAS of Satisfaction than patients treated within a week from injury (Table 4). The distribution of SEFAS in these patient groups are presented in Figure 2.

**Table 4.** PROMs at Follow-up Stratified by Treatment Within or More Than 1 Week From Injury.

	≤ 7 d, Mean (SD) (n = 86)	>7 d, Mean (SD) (n = 44)	*P* Value^a^
SEFAS	38 (9)	34 (10)	.01
RAND-36^b^	74 (20)	71 (18)	.4
VAS of Pain^c^	2 (2)	3 (2)	<.01
VAS of Satisfaction^d^	8 (2)	7 (3)	.02

Abbreviations: PROMs, patient-reported outcome measures; SEFAS, Self-Reported Foot and Ankle Score; VAS, visual analog scale.

a Post hoc analysis for between-group differences were performed with the Tukey honestly significant difference and Bonferroni tests.

b RAND-36 is a generic PROM for quality of life.

c VAS of Pain: 0 = no pain and 10 = worst possible pain. Pain score is an average value of pain experienced the last 2 weeks before the clinical examination.

d VAS of Satisfaction: 0 = very disappointed and 10 = very satisfied with the result.

After stratifying the patients into 3 groups, the patients in group 3 (>7 days) had a lower mean SEFAS than patients in groups 1 and 2 (Table 5). Linear modeling of SEFAS by time from injury to definitive operation adjusted for age, gender (female), and American Society of Anesthesiologists classification showed that time to operation (*P* = .002) and gender (female) (*P* = .001) were associated with poorer SEFAS (Table 6). Group 3 had a significantly lower SEFAS compared with both group 1 (*P* = .015) and group 2 (*P* = .021) when analyzed in a general linear model with time from injury to operation stratified to the 3 groups as an ordinal variable (Table 6). Quality of life (RAND-36) and satisfaction (VAS) was similar between the 3 stratification groups (Table 5). VAS of pain (*P* = .03) was not significant at α = 0.017 (Table 5).

**Table 5.** PROMs at Follow-up Stratified by Time From Injury to Definitive Surgery.

	<1 d, Mean (SD) (n = 44)	1-7 d, Mean (SD) (n = 42)	>7 d, Mean (SD) (n = 44)	*P* Value^a^
SEFAS	38 (9)	38 (9)	34 (10)	0.04
RAND-36^b^	77 (19)	71 (20)	71 (18)	0.3
VAS of Pain^c^	2 (2)	2 (2)	3 (2)	0.03
VAS of Satisfaction^d^	8 (2)	8 (2)	7 (3)	0.06

Abbreviations: PROMs, patient-reported outcome measures; SEFAS, Self-Reported Foot and Ankle Score; VAS, visual analog scale.

a Post hoc analysis for between-group differences were performed with the Tukey honestly significant difference and Bonferroni tests. Group 3 (<7 days) had a mean 1.1 points (95% CI, –2.6, 2.2) higher VAS of Pain than group 1 (<1 day), with a *P* value = .03. However, the result was not significant at an alpha level of .017.

b RAND-36 is a generic PROM for quality of life.

c VAS of Pain: 0 = no pain and 10 = worst possible pain. Pain score is an average value of pain experienced the last 2 weeks before the clinical examination.

d VAS of Satisfaction: 0 = very disappointed and 10 = very satisfied with the result. Siginificance level for SEFAS is .05 and .05/3 = .017 for RAND-36, VAS of Pain, and VAS of Satisfaction.

**Table 6.** General Linear Model With Univariate Analysis of Variance of SEFAS With Time From Injury to Operation, Adjusted for Age, Gender (Female), and ASA Classification.^a^

Parameter	Beta	SE	*t*	Significance Level	95% CI	
					Lower Bound	Upper Bound
Time from injury to operation (days) as a continuous variable (*R*-squared = 0.153)						
Intercept	42.43	3.25	13.05	<.001	35.99	48.86
Time from injury to operation (days)^b^	−0.45	0.15	−3.09	.002	−0.73	−0.16
Gender (female)	−5.79	1.73	−3.35	.001	−9.22	−2.37
Age, y	0.08	0.05	1.56	.12	−0.02	0.17
ASA classification	−2.13	1.43	−1.49	.14	−4.96	0.69
Time from injury to operation (days) as a categorical, ordinal variable (3 groups) (*R*-squared = 0.142)						
Intercept	37.72	3.48	10.85	<.001	30.84	44.60
Time from injury to operation						
Group 1 (<1 d)	4.63	1.88	2.46	.015	0.90	8.35
Group 2 (1-7 d)	4.41	1.89	2.34	.02	0.67	8.15
Groups 3 (>7 d)	0^c^					
Gender (female)	−5.48	1.74	−3.15	.002	−8.92	−2.03
ASA classification	−2.55	1.44	−1.78	.08	−5.39	0.29
Age, y	0.07	0.05	1.47	.15	−0.03	0.17

Abbreviations: ASA, American Society of Anesthesiologists classification; SEFAS, Self-Reported Foot and Ankle Score.

a Results of analyses with time to operation as both a continuous variable and a categorical, ordinal, variable.

b The continuous variable of time from injury to operation was used in this analysis.

c Reference group.

Mean SEFAS at follow-up for patients who were treated with a temporary external fixation prior to definitive surgery was 33 (SD 10) compared to 38 (SD 8) for patients that did not get an external fixator, *P* = .005 (Table 7). The 5 patients treated with temporal external fixator due to severe soft tissue swelling and blisters had the lowest reported mean SEFAS of 27 (SD 9).

**Table 7.** SEFAS for Patients Treated With or Without a Temporary External Fixator, and Patients With and Without a Dislocation Fracture.

	No Ex-Fix, Mean (SD) (n = 89)	Ex-Fix, Mean (SD) (n = 41)	*P* Value	Patients With Dislocation Fracture (n = 59)		
				<7 d, Mean (SD) (n = 35)	>7 d, Mean (SD) (n = 24)	*P* Value
SEFAS	38 (8)	33 (10)	.005	38 (10)	32 (12)	.05
VAS of Pain^a^	2 (2)	3 (2)	.001	2 (2)	3 (2)	.008
VAS of Satisfaction^b^	8 (2)	7 (3)	.04	9 (2)	7 (2)	.001

Abbreviations: Ex-Fix, external fixator; SEFAS, Self-Reported Foot and Ankle Score; VAS, visual analog scale.

a VAS of Pain: 0 = no pain and 10 = worst possible pain. Pain score is an average value of pain experienced in the last 2 weeks before the clinical examination.

b VAS of Satisfaction: 0 = very disappointed and 10 = very satisfied with the result.

Fracture type (Weber B and C) and patients with and without a dislocation fracture reported similar score on subanalyses of SEFAS (*P* = .6 and *P* = .4, respectively). Mean SEFAS among patients who had a dislocation fracture of the ankle and who were treated within a week from injury was 38 (SD 10) points and 32 (SD 12) points for those treated after a week from injury, *P* = .05 (Table 7).

The mean difference in dorsal flexion at follow-up between the injured and noninjured ankle was similar between patients treated within a week (9 degrees [SD 9]) and patients treated later than a week from injury (11 degrees [SD 8]), *P* = .16. It was also similar across the 3 stratification groups: 9 degrees (SD 10) in group 1, 9 degrees (SD 7) in group 2, and 11 degrees (SD 8) in group 3, *P* = .37.

### Complications

Applying a dichotomous analysis of complications comparing patients treated within a week from injury to patients treated after a week from injury revealed similar frequencies of FRI (*P* = .83), postoperative soft tissue problems (*P* = .34), nerve injury (*P* = .12), and reoperations (*P* = .45). However, patients treated later than a week from injury had more frequent preoperative soft tissue problems (<.001) and high-grade osteoarthritis (.02). Implant removal was more frequent among patients treated within a week from injury (*P* = .002).

A total of 9 patients (7%) needed revision surgery; there was no difference in reoperation between the 3 stratification groups (Table 8).

**Table 8.** Distribution of Complications Based on Time From Injury to Operation.^a^

	<1 d, n (%) (n = 44)	1-7 d, n (%) (n = 42)	>7 d, n (%) (n = 44)	*P* Value
Complications				
Fracture-related infection	9 (20)	8 (19)	8 (18)	>.99
Soft tissue problems preoperatively	0	2 (5)	10 (23)^b^	.001
Soft tissue problems postoperatively	3 (7)	9 (21)	9 (20)	.10
Nerve injury	7 (16)	5 (12)	11 (25)	.30
Reoperations	5 (11)	2 (5)	2 (4)	.40
Implant removal	23 (52)^c^	14 (33)	7 (16)	.02
Osteoarthritis grade 2-4	3 (7)	5 (12)	11 (25)	.05

a Removal of syndesmotic screws were part of the treatment protocol and is the cause of removal for 11 of 23 patients in group 1 (<1 day). Preoperative soft tissue problems were severe swelling and bullae development. Postoperative problems were prolonged wound healing, skin necrosis, and wound secretion. Patients included in fracture-related infections had either prolonged wound healing, wound discharge/secretion, or wound dehiscence, and does not include skin necrosis that were not surgically treated. Post hoc analysis of between-group differences of categorical variables were performed with Bonferroni method for adjusting *P* values while comparing column proportions.

b Statistically significant difference at an alpha level of 0.05 between group 3 and both group 1 and 2, but not between groups 1 and 2.

c Statistically significant difference at an alpha level of 0.05 between group 1 and both group 2 and 3, but not between groups 2 and 3.

Clinical signs of FRI were found in 25 (19%) of the 130 patients, with no difference between the groups (Table 8). Comparing patients with FRI to those without FRI within group 3, a tendency toward lower mean SEFAS at follow-up was seen in patients with FRI (27 [SD 12] vs 35 [SD 9]; *P* = .07).

Nerve injuries were present—either as a reduced skin sensation or paresthesia on the dorsolateral side of the foot—in 23 of 130 (18%) patients. The incidence was similar across the 3 groups.

Planned removal of syndesmotic screws was the main reason for a high frequency of implant removal in group 1 (11 of the 23 patients).

## Discussion

A major finding in this study was that patients with severe ankle fractures waiting >7 days until definitive surgery reported lower patient-reported outcome, a lower VAS of Satisfaction, and a higher VAS of Pain than to those who had definitive surgery within a week from injury. Those who waited more than a week had more frequently received temporary external fixation prior to definitive surgery and had a longer duration of surgery. Gender (female) was also an independent risk factor for worse patient-reported outcome, similarly to Storesund et al,^28^ who found women to report a higher postoperative VAS of Pain than men.

Lower patient-reported outcome and more pain in patients who had definitive surgery after a week from injury is also reported by others.^22,26^ Normative mean values for SEFAS are 46 (SD 5) for men and 42 (SD 6) for women, and the minimal clinically important difference has formerly been described to be 5 points.^7,9^ At a mean 26 months, patients in all 3 stratification groups in the current study reported an SEFAS that was more than 5 points lower than that found in the general population among men and 4 points lower than among women, reflecting the severity of their injury. However, the use of minimal clinically important difference or minimal clinically important change (MCIC) is intended for interpretation of the treatment effect within individual patients. To apply these cutoffs as a yardstick on a group level is warned against and even termed misleading.^3,17^ Further research is needed to aid the interpretation of ankle-specific PROMs, including further estimates of minimal important differences between treatment groups. Based on limited available evidence, we believe that there is an important difference between the 2 groups (treatment within or after a week from injury). The histogram of SEFAS among patients treated after 1 week from injury shows that the majority of these patients report below 35 points while patients treated within 1 week from injury are clustered above 35 points. Fractures that are difficult to reduce might suggest more extensive and complex fractures, but the patient and fracture characteristics and injury mechanisms were similar, regardless of time between injury and definitive surgery. The tendency of more frequent high-grade osteoarthritis in patients who waited more than a week until definitive surgery may explain the inferior ankle function found among these patients (*P* = .05). Also, neither the presence of a dislocation fracture or the fracture type had an association to lower PROM score. However, patients with a dislocation fracture who were treated within a week from injury reported better PROM than those treated after a week from injury. Loss of dorsiflexion has formerly been reported to predict a poor clinical outcome,^13^ but no such association was found in the present study.

Patients who were treated with a temporal external fixator reported poorer clinical outcome. The use of temporal external fixator was an important contributor to the prolonged length of stay. For some of the patients the external fixator was applied several days after admission and thus further prolonging the time to definitive operation. After application of the external fixator, there could be some complacency—the ankle would be considered safe and not in need of urgent surgery, further postponing definitive surgery. Further, as a consequence of the use of temporary external fixation, the patients were exposed to surgery twice—with the risks of complications that might follow. It has been suggested that the temporal external fixator could be placed in the emergency department in local anesthetics and sedation to reduce delay till definitive surgery.^27^ This is, however, not common practice at our clinic. The application of a temporal external fixator to reduce soft tissue complications and improve outcome did not seem to benefit the patients in the present study. Almost 80% of patients who had definitive surgery more than 1 week from the time of injury got a temporary external fixation. Due to sparse data for the patients who had external fixation and definitive surgery within 1 week from injury, and those without external fixation with definitive surgery after 1 week from injury, subanalyses of SEFAS on these groups would have limited value. With the current data, it is not possible to distinguish between the impact of external fixation and time from injury to operation on SEFAS. Patient characteristics and fracture type (Weber B/C) did not differ between those who did not and those who did get an external fixator. In addition, the majority of patients with a dislocation fracture did not get a temporary external fixator. Even among patients with a dislocation fracture, those treated within a week from injury had the highest mean SEFAS. All in all, acute, definitive surgery would reduce both the use of external fixation and time until definitive surgery.

The longer duration of surgery among patients who waited more than a week till ORIF might reflect a more complex injury or operation. The fact that three-quarters of these patients were treated with a posterior approach also supports such a notion. Mean time to definitive operation was 12 days among patients who waited more than a week. Such a delay could make the soft tissue and fractures more challenging to handle intraoperatively compared to at an immediate operation—leading to a more meticulous surgical procedure. Poorer outcome in this group, compared to those with a shorter wait, is similar to the findings of other studies.^22,25^

Similar rates of FRIs were found between the 3 groups of the current study with an overall rate of 19%.^21^ All groups had a higher complication rate than the 12.9% that Schepers et al found in patients operated later than 6 days from injury and 3.4% in patients treated within 6 days from injury.^26^ The same authors also reported inferior clinical outcomes in patients with infectious wound complications, similar to the current study. Saithna et al also report higher incidence of infection in patients treated after 6 days from injury (3.6% vs 20.7%, *P* = .010).^25^ The high rate of postoperative infection in the present study was a worrying finding that requires further investigation.

SEFAS was chosen as the primary outcome, as it is validated for ankle fractures. This strengthens the reliability of the current results. Among several questionnaires, SEFAS has been considered to have the best measurement properties for patients treated for ankle fractures.^12^ The current cohort was also evaluated with a multitude of outcome measures, including radiographs. Further, a focus on a thorough reporting of complication rates has allowed for subanalyses across the stratified groups of the study. In summary, this gives a more complete picture of outcomes and functional performance after severe ankle fractures. The current study presents a transparent evaluation of clinical practice at a Level 1 trauma hospital.

The retrospective study design limits the generalizability of the current results. Seventy-two percent (130/180) of the eligible patients were examined. A level of nonresponder bias may therefore be present. Similarly, although fracture characteristics are similar between the groups, a selection of more severe injuries to group 3 cannot be ruled out. This may confound our findings to some extent. In addition, there may have been an interaction between the use of temporary external fixation and time to surgery, as discussed above. The majority of patients who waited more than 7 days until definitive surgery had their ankle fracture treated via a posterior approach.

## Conclusion

In our study, we found that patients with low-energy ankle fractures with a posterior malleolar fragment who waited more than a week for definitive surgery had a higher rate of preoperative soft tissue problems and reported poorer clinical outcome and more pain. The patients with delayed treatment were more often treated with a temporary external fixation before definitive surgery. In our series, use of temporary external fixation to resolve soft tissue problems preoperatively did not prevent poorer ankle function 2 years after surgery. A delay from injury until definitive surgery of more than 7 days was not found beneficial for the patients included in this study. Our findings further suggest that patients with dislocation fractures had better outcomes when definitively treated within 7 days.

## Supplemental Material

sj-pdf-1-fai-10.1177_10711007211070540 – Supplemental material for Association of Delayed Surgery for Ankle Fractures and Patient-Reported Outcomes Click here for additional data file. Supplemental material, sj-pdf-1-fai-10.1177_10711007211070540 for Association of Delayed Surgery for Ankle Fractures and Patient-Reported Outcomes by Kristian Pilskog, Teresa Brnic Gote, Heid Elin Johannessen Odland, Knut Andreas Fjeldsgaard, Håvard Dale, Eivind Inderhaug and Jonas Meling Fevang in Foot & Ankle International
